# THE ALCOHOL CONSUMPTION IS AMENDED AFTER BARIATRIC SURGERY? AN INTEGRATIVE REVIEW

**DOI:** 10.1590/0102-672020180001e1378

**Published:** 2018-07-02

**Authors:** Valeria Duarte GREGORIO, Roselma LUCCHESE, Ivânia VERA, Graciele C. SILVA, Andrecia SILVA, Rayrane Clarah Chaveiro MORAES

**Affiliations:** Universidade Federal de Goiás , Goiânia, GO, Brazil

**Keywords:** Substance-related disorders, Bariatric surgery, Alcoholism., Transtornos relacionados ao uso de sustâncias, Cirurgia bariátrica, Alcoolismo

## Abstract

***Background*:**

Bariatric surgery has been an alternative when conservative methods of weight loss fail. Patients undergoing bariatric surgery have an increased risk of up to 6.5% of problems related to alcohol.

***Objetive*:**

To review the literature about the changes on alcohol consumption in this public.

***Method*:**

Database was accessed from June of 2015 to January of 2016 by searching “bariatric surgery” AND “alcoholism”, and their Portuguese equivalents. Science Direct, PubMed, Lilacs and Medline, besides manual search, were searched. To be included, the paper should have been published between 2005-2016 and related to bariatric surgery and alcoholism. Theses, dissertations, unpublished papers, case reports and theoretical studies were excluded. In 2005 there was only one review of change in alcohol metabolism in patients undergoing bariatric surgery. There were no publications in 2006. In 2007, only one study was published, and it did not meet the inclusion criteria. In 2010, there was an increase of 13% in publications and of 20% in 2012, reaching 40% in 2013.

***Conclusion*:**

The prevalence and incidence of alcohol consumption in relation to the postoperative time was six months to three years with higher incidence in men. Roux-en-Y gastric bypass showed greater association with increased alcohol consumption during the postoperative period. This and other studies showed that the pattern of alcohol consumption is important to be faced as a problem in bariatric surgery follow-up.

## RESUMO

***Introdução*:**

A cirurgia bariátrica tem-se mostrado alternativa para o insucesso dos métodos conservadores de emagrecimento. Pacientes submetidos à ela têm aumento do risco de 6,5% de problemas relacionados ao álcool.

***Objetivo*:**

Realizar revisão integrativa para verificar alteração do consumo de álcool neste público.

A base de dados Science Direct, PubMed, Lilacs, Medline e busca manual foram acessadas entre os meses de junho de 2015 a janeiro de 2016 com os descritores “cirurgia bariátrica” e “alcoolismo” e equivalentes em inglês Os critérios de inclusão foram publicações entre junho de 2005 a janeiro de 2016, relacionadas à cirurgia bariátrica e ao consumo de álcool. Foram excluídas teses, dissertações, trabalhos não publicados, relatos de casos e estudos teóricos.

***Resultado*:**

No ano de 2005 houve somente uma revisão relacionada à alteração do metabolismo do álcool em pacientes submetidos à cirurgia bariátrica. Não houve publicações em 2006. Em 2007, houve uma publicação fora dos critérios deste trabalho. Em 2010 observou-se aumento de publicações em 13% e 20% em 2012, atingindo 40% em 2013.

***Conclusão*:**

A prevalência e a incidência do consumo de álcool em relação ao tempo de pós-operatório foram de seis meses a três anos com maior incidência em homens. O bypass gástrico em Y-de-Roux apresentou maior associação ao aumento do consumo de álcool durante o pós-operatório. Este e outros estudos mostraram que o consumo de álcool se mostrou importante e deve ser enfrentado como potencial problema no seguimento em longo prazo após operação bariátrica.

## INTRODUCTION

About 3.4 million adult deaths annually with cases of obesity, and the prevalence of adult obesity is 11% globally and 35% in the United States^1^

In cases in which patients do not show positive responses to conventional weight loss attempts as diet, physical activity and drug therapy, surgery has been taken into account, more precisely the bariatric surgery (BS)^24^.

Aiming to better health conditions, and following strict standards to be performed, the BS has been an alternative and an effective treatment for morbid obesity, in cases that body mass index ≥40 kg/m^2^ or ≥35 kg/m^2^ ^4^ with associated comorbidities (diabetes, sleep apnea, hypertension, dyslipidemia, coronary heart disease and osteoarthritis), failure of well conducted conservative weight loss methods, and absence of alcohol consumption and psychiatric disorders^21^

A situation to be observed before and after the surgery that calls more attention is the alcohol consumption in patients undergoing BS for weight loss^11^ ^,^ ^16^, which can increase the risk of developing problems related to alcohol abuse in up to 6.5%^26^.

Among the problems, we can highlight the possibility of transferring compulsive eating to alcohol abuse^22^ ^,^ ^23^. The prevalence rates indicate an increase of 7.6% to 9.6% in 12 months after BS^15^. Studies also show that there is no intention in correcting the problematic^2^ ^,^ ^16^.

The use of this psychoactive substance prevents regular glycemic control^5^ and causes poisoning and changes with less dosage, compared to the period before the surgery^15^, being considered a challenge in the rehabilitation process^15^.

Considering this problematic for research, the need for an integrative review on alcohol consumption in patients undergoing BS come to light, with the following guiding questions: “Does the pattern of alcohol consumption change in patients undergoing BS?”; “What do the investigations about this subject reveal?” 

The objective of this review was to summarize the scientific knowledge produced on the pattern of alcohol consumption in patients undergoing BS between the years 2005 to 2015.

## METHOD

This is an integrative review^20^ guided by the question of alcohol abuse among individuals who have undergone BS, inquiring about the change or not in the pattern of this substance use in this population. Therefore, Science Direct, PubMed, Latin American and Caribbean Center on Health Sciences Information (LILACS), and Medical Literature Analysis and Retrieval System Online (MEDLINE®) were accessed.

The search in the databases was conducted between June and August 2015 simultaneously by two researchers. We used the terminology adopted by Health Sciences Descriptors (Decs) and Medical Subject Headings (Mesh), identifying the headings in English and Portuguese version “bariatric surgery” AND “alcoholism”. Later, with the selected items, we proceeded with a manual search (hand-search) in their references.

The inclusion criteria for the selection of manuscripts were: results of research that addressed the theme, that is, the relationship between alcohol consumption in patients undergoing BS; field investigations, as original articles and short communication; publications between the years 2005 to 2016 in English, Spanish and Portuguese. Theses, dissertations, unpublished papers, case reports and theoretical studies were excluded, as well as manuscripts that were repeated in databases.

The studies were organized in Excel 2007® with records of information guided by the data collection instrument: title, author/year, journal, year of publication, objective, study design, population, level of evidence and main results and conclusions found.

The level of evidence was assigned according to the classification by study design in seven categories^24^: level 1, for systematic reviews and meta-analysis of relevant randomized controlled clinical trials or derived from clinical guidelines based on systematic reviews of randomized controlled trials; level 2, for evidence derived from at least one randomized controlled clinical trial and well-designed; level 3, for evidence from well-designed clinical trials without randomization; level 4, for evidence of cohort studies and well-designed case-control; level 5, for evidence of systematic review of descriptive and qualitative studies; level 6, for evidence from a single descriptive or qualitative study; and level 7, for evidence from officials opinion and/or expert committees report^19^.

After this step, the observational analysis was carried out, with assessment of the main types of studies and observation of the relationship between the alcohol consumption and the type of procedure performed.

A database was built with information about changes in the pattern of alcohol consumption and substance abuse in patients undergoing BS, in order to facilitate access to major developed research on this topic

## RESULTS

In Pubmed were initially found 33 articles and after observational analysis were selected 10. The themes addressed by the articles were: characterization of the prevalence of alcohol consumption pre and postoperative check with the independent predictors; description of the disorder phenotypes by alcohol abuse by the AUDIT and analysis of the relationship between the degree of weight loss the incidence of AUDIT.

In Science Direct database were initially selected 53 articles and after observational analysis were selected four articles that met the requirements of this review. The issues addressed highlighted the verification of the change in alcohol metabolism after bypass; characterization of alcohol consumption pre and postoperative follow-up for two years of surgery and determination of the associated factors and absorption of alcohol before and after laparoscopic sleeve gastrectomy.

In the Lilacs and Medline databases were found respectively four and 15 articles, but the items were duplicate.

In the manual search two articles were selected. The themes addressed the prospective evaluation of the relationship between the consumption of alcohol and smoking in patients before and after bariatric surgery through the AUDIT and analyze the sensitivity of patients to alcohol consumption after surgery as well as changes in the pattern of consumption during the operative post bariatric surgery.

In total were selected 16 articles describing the title, objectives, level of evidence, instruments used, number of participants and the main aspects and results of each study.

**FIGURA 1:**
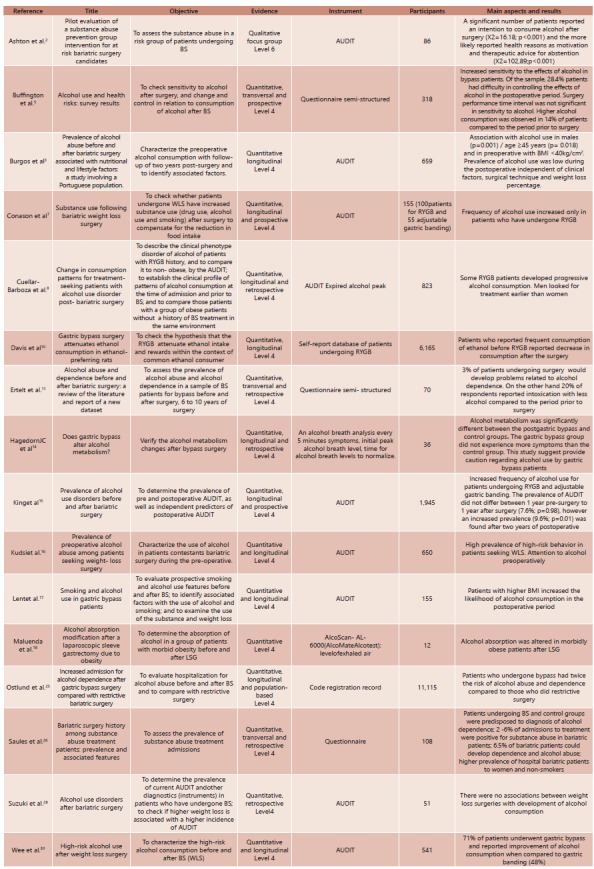
Os estudos publicados entre 2005 e 2016 sobre o tema

Figure 1 shows the main studies in the past 10 years relating to bariatric surgery to alcohol consumption with emphasis in relation to the objectives, scientific evidence, the instruments used in the studies, number of participants and main characteristics and results found.

BS=cirurgia bariátrica; AUDIT=Alcohol Use Disorders Identification Test; WLS=cirurgia para perda de peso; RYGB=bypass gástrico Y-de-Roux; IMC=índice de massa corporal; LSG=gastrectomia vertical laparoscópica

In 2005 there were just one review of alcohol absorption and metabolism in bariatric surgical patients. In 2006 there were no publications^6^. In 2007 we found a quantitative study that suggested provide caution regarding alcohol use by gastric bypass patients^14^. In 2009 was found one interventional observational study, but it was not included in this i review because it was a fieldwork^9^. From 2010, the number of publications increased with prevalence of 13%^18^ ^,^ ^26^ and this increase continued in 2012 with 20% of publications^10^ ^,^ ^15^ ^,^ ^28^. The year with the highest percentage of publications was 2013, with a prevalence of 40%^2^ ^,^ ^7^ ^,^ ^16^ ^,^ ^17^ ^,^ ^23^ ^,^ ^29^.

From 2013 there was an increase in the number of longitudinal studies (level of evidence IV), in order to respond, with more robust methodologies and greater scientific nature, the changes in the pattern of consumption, the association to the type of bariatric procedure, and attitudes regarding the reduction of alcohol consumption during the postoperative period^3^ ^,^ ^7^ ^,^ ^8^ ^,^ ^16^ ^,^ ^17^ ^,^ ^23^.

## DISCUSSION

Studies regarding the use of psychoactive substances in patients undergoing CB showed limitation about the sample size, as 53% of the studies analyzed showed an average of 90 participants^2^ ^,^ ^7^ ^,^ ^11^ ^,^ ^17^ ^,^ ^18^ ^,^ ^26^ ^,^ ^27^ ^,^ ^28^ in addition to the restriction of not being considered probabilistic or population-based samples. Five studies were guided by retrospective data collection, which can interfere with the quality of information that, in turn, is dependent on the quality of previous records^8^ ^,^ ^11^ ^,^ ^26^ ^,^ ^28^ ^,^ ^29^.

Likewise, methodological limitations were observed: 33% of the studies were cross-sectional type, which makes the causality between effect and exposure/impact^5^ ^,^ ^11^ ^,^ ^18^ ^,^ ^26^ ^,^ ^28^. However, relevant associated factors for the production of knowledge of this subject could be observed.

At the same time, 43% of studies presented robust epidemiological methods, such as the longitudinal ones^7,8,10,15,16,17,23,29^ and one with population-based sample^23^.Still considering the methods, we highlight the existence of one qualitative study^2^.

Taking into account the surgical indications and in accordance to the objectives of this review, we have intended to list the high risk criteria that contraindicate the procedure, through the guidelines of American Society for Metabolic and Bariatric Surgery. Abusive history of psychoactive substances; regular use of alcohol pre-surgery; the realization of the Roux-in-Y gastric bypass (RYGB) and smoking^21^ are some of these criteria.

Among the high-risk criteria, checking the real prevalence of alcohol abuse during the postoperative period has been observed^5^ ^,^ ^11^ ^,^ ^15^ ^,^ ^16^ ^,^ ^28^. There is evidence that 3.0%^11^ of individuals undergoing the surgery will develop problems resulting from the use of alcohol. At the same time, we observe an incidence of alcohol consumption of 4.9%^8^ and a 6%^11^ to 6.5% variation of prevalence in the postoperative period^26^.

Considering the pattern of alcohol consumption during the postoperative period, changes in this period are described^5^ ^,^ ^11^ ^,^ ^18^ ^,^ ^26^ ^,^ ^28^. There is a correlation between the use of alcohol with time after surgery, with an increase of 2% of alcohol consumption rate in two years in the postoperative^15^.

The postoperative time relationship was also described. Study found a lower prevalence of substance use in the period up to six months after surgery, while those who had a higher intake of alcohol were in over one year of the procedure. This can be justified by the discouraging alcohol consumption in the prior period to six months^5^.

With reference to the change in the pattern of alcohol use, its increase has been reported in 33% of cross-sectional studies selected in this review^7^ ^,^ ^15^ ^,^ ^16^ ^,^ ^23^ ^,^ ^28^. On the other hand, in 13% of the studies, more precisely in the longitudinal studies^10^ the reduction of alcohol consumption after BS was estimated with a decrease of 9.1%^17^.

Two studies addressed the reduction of alcohol use after weight reduction surgery referring to patients submitted to RYGB^10^ ^,^ ^14^. However, these are individuals or database studies, which contained only patients who were submitted exclusively to such surgical procedure. In this review, 40% of the studies investigated patients undergoing various techniques and showed different results, that is, we identified an increase in alcohol consumption in the postoperative with the RYGB procedure^7^ ^,^ ^8^ ^,^ ^15^ ^,^ ^23^ ^,^ ^27^ ^,^ ^28^.

Other findings observed were related to hypoglycemic episodes, due to the reduced availability of glucose, by suppressing gluconeogenesis, a situation that gets worse with alcohol consumption^5^. Individuals are more sensitive to the effects of alcohol^5^ ^,^ ^23^ ^,^ ^27^ resulting in intoxication because of the quantity of alcohol ingested after BS^11^. We also noted an increasing prevalence of hospitalizations resulting from alcohol consumption, with men seeking more treatment compared to women^11^. In contrast, a higher prevalence of hospitalization in female and nonsmokers bariatric patients was also observed^26^.

Other remarks are about the possibility of transferring eating to alcohol consumption, which would strengthen the dependency status of this substance^22^ ^,^ ^23^. Patients with high body mass index are more likely to develop alcohol consumption during the postoperative period^17^. Also the weight loss appears as a risk factor for the consumption of alcohol during postoperative^13^.

The instruments used in the integrative review to verify the consumption of alcohol were Alcohol Use Disorders Identification Test (AUDIT)^2^ ^,^ ^3^ ^,^ ^7^ ^,^ ^8^ ^,^ ^15^ ^,^ ^16^ ^,^ ^17^ ^,^ ^28^ ^,^ ^29^ with a prevalence of 53.3%; the Self-Report Questionnaire^5,10,11,26^ with 26.6%; and the level of exhaled air with AlcoScan - AL-6000 (AlcoMateAlcotest)^18^ with 6.6%, as well as records^23^, with 6.6% of prevalence. Interventional observational analysis was also performed, representing 6.6% of the studies^27^.

AUDIT^4^, originally developed as a collaborative project of the World Health Organization in the late 1980s and validated in Brazil in 1999, is configured today as one of the measures employed worldwide for early detection screening of risk to the harmful use of alcohol. The same applies to the tracking of alcohol abuse in clinical samples and the general population^12^ ^,^ ^25^.

The AUDIT was used in different ways in these studies. Some applied this tracker before and after the surgical procedure^15^ ^,^ ^17^ ^,^ ^29^; others, at the time of data collection, generated risk estimation or dependence at the time of the survey^7^ ^,^ ^8^ ^,^ ^28^. Finally, the AUDIT was applied comparing bariatric patients with non-obese individuals suggesting the realization of a preventive AUDIT in patients who should undergo RYGB^8^.

We emphasize the importance of pre-operative advice regarding the consumption of alcohol as a protective factor against the risk of alcohol abuse^16^. We should let the patients know about the adverse effects of alcohol, with the intention of reducing the consumption during the postoperative period. These studies also observed that the search for improvement in health figured as a motivation to reduce alcohol consumption^7^ ^,^ ^16^.

All studies analyzed generated variables for future research and raised the issue surrounding the weight loss process and health problems in particular with the use of alcohol.

## CONCLUSIONS

In principle, we found differences in postoperative period and gender related to the use of alcohol. The prevalence and incidence of alcohol consumption had a variation of about six months to three years in postoperative period. As to gender, both sexes were involved, but there was a higher incidence of men seeking treatment because of alcoholic substance use. 

However, there was no consensus about sensitivity to the use of alcohol, which increases after the BS. Smaller alcoholic doses cause greater toxicity, compared to the period prior to the surgery. Likewise, research studies have addressed more frequently studies on BS that used RYGB technique, because this is the most usual procedure for the surgical treatment of obesity, indicating the need for comparative studies with other common techniques. .

Another relevant synthesis relates to the transfer of food cravings for alcohol consumption; however, it was found that this dimension has been discussed theoretically.
